# Prevalence and risk factors of childhood anemia in Nepal: A multilevel analysis

**DOI:** 10.1371/journal.pone.0239409

**Published:** 2020-10-06

**Authors:** Mohammad Rocky Khan Chowdhury, Md. Mobarak Hossain Khan, Hafiz T. A. Khan, Md. Shafiur Rahman, Md Rashedul Islam, Md Moinul Islam, Baki Billah

**Affiliations:** 1 Department of Public Health, First Capital University of Bangladesh, Chuadanga, Bangladesh; 2 Department of Social Relation, East West University, Dhaka, Bangladesh; 3 College of Nursing, Midwifery and Healthcare, University of West London, London, United Kingdom; 4 Research Center for Child Mental Development, Hamamatsu University School of Medicine, Japan; 5 Department of Global Health Policy, Graduate School of Medicine, University of Tokyo, Tokyo, Japan; 6 School of Business, Middlesex University, London, United Kingdom; 7 Department of Epidemiology and Preventive Medicine, School of Public Health and Preventive Medicine, Monash University, Melbourne, Australia; Anglia Ruskin University, UNITED KINGDOM

## Abstract

**Introduction:**

Anemia is a common problem in children particularly in developing countries and taking steps to tackle it is one of the major public health challenges for Nepal. The objective of this study is to investigate the prevalence of individual, household and community level determinants of childhood anemia in Nepal.

**Methods:**

Data was taken from a nationally representative sample of 1,942 Nepalese children aged from 6–59 months. The Chi-square test was used to determine the bivariate relationship between the selected variables and childhood anemia and a multilevel logistic regression model with a random intercept at household and community level was used to identify important determinants of this kind of anemia.

**Results:**

The results showed that 52.6% (95% CI: 49.8%-55.4%) of the children were anemic while 26.6% (95% CI: 24.0%-29.3%) of them were moderate to severe. The prevalence of overall anemia was higher among children aged less than 11 months as well as in underweight children, children of underweight, anemic and uneducated mothers and those in the terrain ecological regions. Multivariable analysis showed that children aged less than 11 months, who were underweight and had anemic mothers were more likely to have moderate or severe anemia. Children in the hilly ecological region were less likely to have it compared to mountain and terrain ecological regions. Children in middle-class families and children of mothers who completed secondary education were more likely to have anemia.

**Conclusion:**

Nepal is facing a serious public health problem due to the high prevalence of childhood anemia. This adverse situation occurs due to socio-demographic and geographical factors such as age, malnutrition status, mother’s anemia status, socio-economic status and regional variations. Prevention of childhood anemia should be given top priority in Nepal and should be considered as a major public health intervention.

## Introduction

Childhood anemia is a major nutritional problem throughout low- and middle-income countries, particularly those in Africa and Asia where it comprises around 45% of cases [1]. In 2016, the estimated prevalence was approximately 55% in South Asia and 60% in Sub-Saharan Africa [1]. The World Health Organization (WHO) has estimated that globally, over 293 million children under five are anemic, with a prevalence of around 47.4% [2]. All forms of anemia, particularly a severe form of it, may cause permanent cognitive damage and impaired psychomotor development in children by decreasing attention span and shortening memory [3]. Iron deficiency anemia accounts for around 60% of all cases of anemia and is also associated with many psychosocial and economic disadvantages that can affect child development [4, 5]. Also, children in younger age groups with severe anemia, caused by malaria and iron deficiency, are at an increased risk of mortality [6, 7]. In 2010, roughly 0.12 million preventable deaths of women and children occurred due to iron deficiency anemia [5]. Anemia should therefore be viewed as a serious public health problem in Nepal with a higher prevalence found among preschool children [8].

Considerable public health achievements have been made in Nepal for reducing maternal and child mortality, but anemia prevention remains a significant challenge [9]. Iron deficiency anemia is one of the common types of malnutrition in the country comprising over 48% of anemic children [2] and is one of the leading causes of childhood morbidity and death [10].

A number of studies on childhood anemia conducted in Nepal looked into the significance of socio-demographic, anthropometric and geographical factors. However, well-documented research on the impact of these risk factors is still scarce [11–13]. Existing studies were unable to draw out the relationships between multilevel factors and childhood anemia due to, for example, involving a limited number of variables and inadequate use of statistical methods [11–13]. Several studies used fixed-effect models, such as binary logistic regression, Poisson models to identify the determinants of anemia but none of them addressed variations at individual, household and community levels for identifying further determinants of moderate to severe childhood anemia [5, 14]. Therefore, the aim of this study was to estimate the prevalence of childhood anemia in Nepal including its determinants with multilevel variations. An understanding of these determinants through a comprehensive study of all the factors would greatly assist in the formulation of policy on this issue in Nepal.

## Materials and methods

### Data

**Fig 1:**
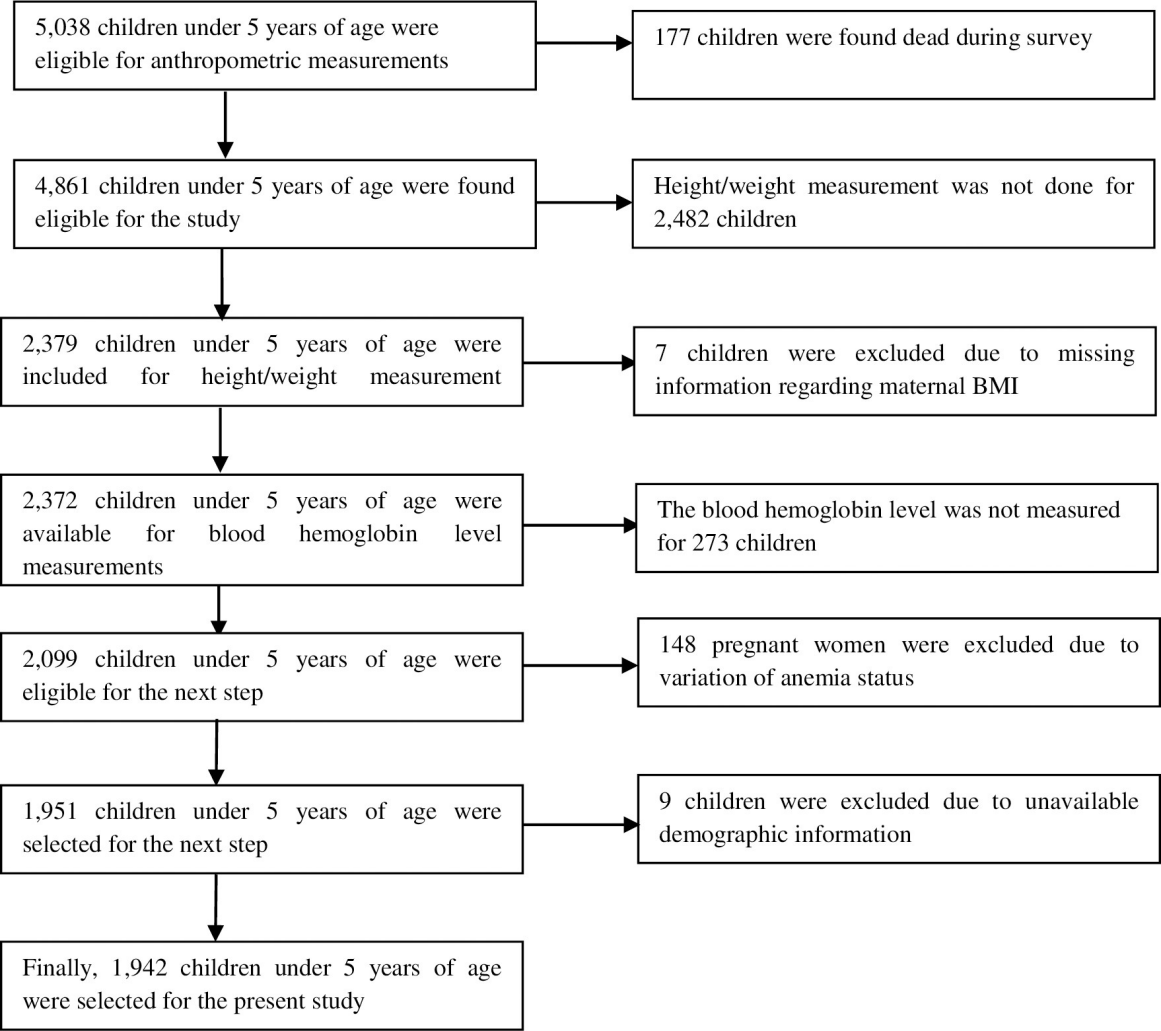
Schematic presentation of sample size selection.

Data was extracted from the 2016 Nepal Demographic Health Survey (NDHS), a nationally representative cross-sectional study conducted from June 2016 to January 2017. This survey was carried out under the aegis of the Ministry of Health and by a Nepali research organization called ‘New ERA’ and had nearly 100 per cent response rate. Technical support was provided by ICF International of Calverton, Maryland, USA and financial support was given by the United States Agency for International Development (USAID). The sample for the 2016 NDHS was drawn from Nepali adults residing in non-institutional dwellings by use of an updated version of a sampling frame that was originally designed for the 2011 National Population and Housing Census (NPHC). There are seven administrative provinces in Nepal with sub-divisions of urban and rural areas that are in turn divided into wards. In rural areas, the wards are small in size each with an average of 104 households, whereas in urban areas each ward has an average of 800 households. The 2016 NDHS samples were selected in two stages in rural areas and in three stages in urban areas. In rural areas, wards were selected as primary sampling units (PSUs) to help select households and in urban areas, one Enumeration Area (EA) was selected form each PSU drawn from a ward, and then households were selected from the sample EAs. The PSUs and EAs were selected using probability proportionate to ward size. In the first stage, a total of 383 wards were selected and in the second stage, one EA was randomly selected from each urban ward. Households were then identified from the selected sampling clusters (either rural wards or urban EAs) and each of those that comprised more than 200 households was selected with probability proportional to segment size. In the final stage, a fixed number of 30 households was selected from each cluster with an equal probability systematic selection. The 2016 NDHS survey identified 5,038 children of which 1,942 aged from 6–59 months were selected for this study (Fig 1 below). This survey was conducted to collect social and-demographic data, health and nutritional data to monitor a wide range of the population. Details of the sampling technique, selection of households, questionnaire and its validation procedure were published elsewhere [15].

### Measuring anemia, outcome variables and independent variables

In the 2016 NDHS, six questionnaires were used involving a household questionnaire, woman’s questionnaire, man’s questionnaire, biomarker questionnaire, fieldworker questionnaire, and verbal autopsy questionnaire. All men aged 15–49 years of age living in every second household were interviewed with a biomarker questionnaire only used in the subsample of households selected for the man’s questionnaire. The biomarker questionnaire was used to record anthropometry measurements, hemoglobin testing, and blood pressure measurement. In these households, women aged 15–49 years and children aged 6–59 months were considered eligible for testing for anemia. Blood specimens for hemoglobin testing were gathered with consent from women and children that involved taking a drop of blood from a finger prick (or a heel prick in the case of children aged 6–11 months) and collected in a microcuvette. Analysis was carried out on-site using a battery-operated portable HemoCue analyzer that could measure the concentration of hemoglobin in the blood. The Nepal Health Research Council and the ICF Macro Institutional Review Board in Calverton, Maryland, USA approved the protocol for the hemoglobin testing [15].

**Fig 2:**
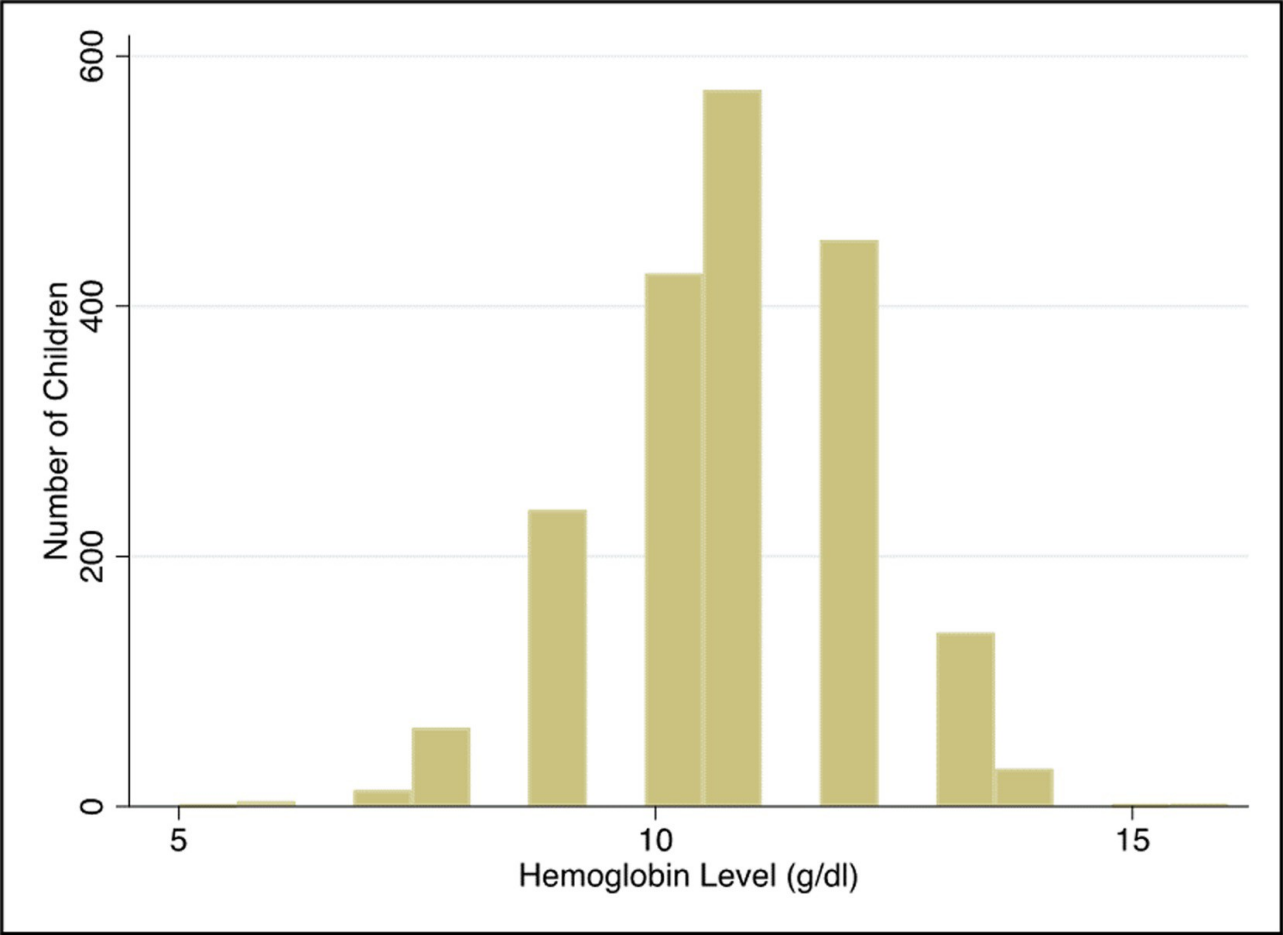
Distribution of hemoglobin level among Nepalese children.

The distribution of hemoglobin levels (gm/dl) is presented in Fig 2 below. A child was considered to be anemic when the estimated hemoglobin level was <11.0 gm/dl. Children were classified as severely (<7.0 gm/dl), moderately (7.0–9.9 gm/dl), and mildly (10.0–10.9 gm/dl) anemic while mothers were classified as anemic (<12.0 gm/dl) according to guidelines provided by the WHO [16].

Socio-demographic, anthropometric and geographical variables of the study were selected based on previously reported studies [5, 14, 17, 18]. Covariates were classified into three characteristic levels: individual, household and community. Individual-level characteristics were child’s age (<11 months, 12–23 months, 24–35 months, 36–47 months, 47–59 months), sex of child (boys, girls), birth order (first, second, third and above), and underweight children (no, yes), mother’s body mass index (BMI) (normal, underweight, overweight), anemia status of mother (non-anemic, anemic) and educational status of mother (no education, primary, secondary and higher). The socio-economic status of households (poorest, poorer, middle, richer, richest) was considered as the household-level characteristics. In demographic and health surveys, the socio-economic status or wealth index has been assessed through principal components analysis of household assets including ownership of durable goods (such as televisions and bicycles) and dwelling characteristics (such as source of drinking water, sanitation facilities and construction materials) [15]. These weighted values were then summed and rescaled to range from 0 to 1 with each household assigned into quintiles as follows: poorest (first quintile); poorer (second quintile); middle (third quintile); richer (fourth quintile) and richest (fifth quintile) [15]. Community level characteristics included place of residence (urban, rural); development region (eastern, central, western, mid-western, far western) and ecological region (mountain, hill, terrain).

### Statistical analysis

Descriptive statistics were presented as percentages where appropriate. Univariate analysis using simple logistic regression and a Chi-square test was used to evaluate associations between outcome (any level of anemia and moderate to severe anemia) and independent variables. In the 2016 NDHS, individuals are nested within households and households are nested within communities, which indicate that individuals and households are not independent of each other [19]. Data of this nature is usually analyzed using a multilevel random intercept model to account for variation in different levels. Therefore, multivariable analysis was performed using a multilevel binary logistic regression model with random intercept at household and community levels. Stata version 11.2/SE (Stata Corp, College Station, Texas, USA) was used for all statistical analysis.

### Ethical statement

The 2016 NDHS was approved by the ethics committee of the Nepal Health Research Council and by the human research ethics committee at ICF Macro International of Calverton, Maryland, USA. Informed consent was obtained from each participant prior to their enrolment and the inclusion of children in the study was only pursued after full consent had been received by either the mother or primary caregiver.

## Results

**Fig 3:**
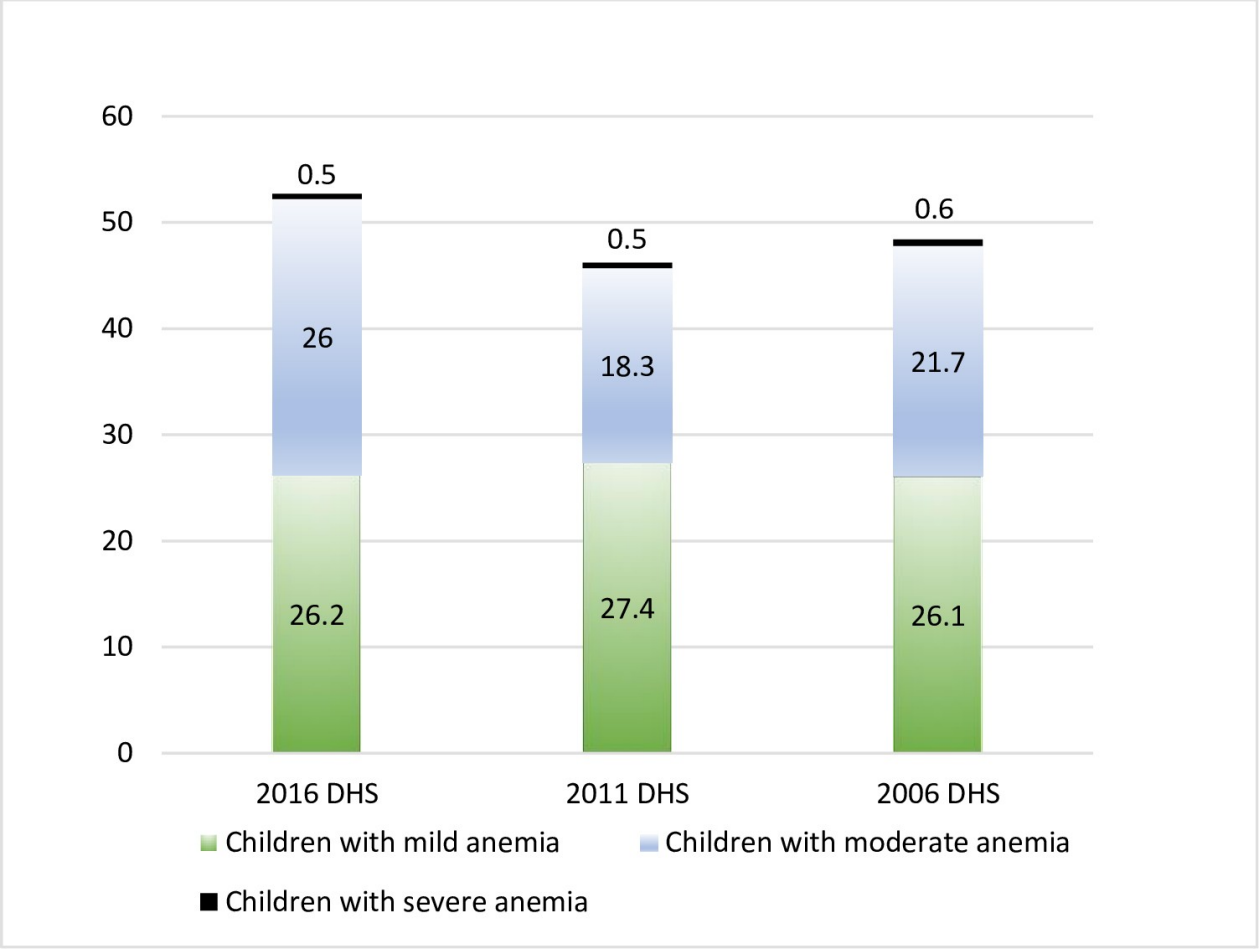
Trend of prevalence for childhood anemia from 2006 to 2016 in Nepal.

The summary statistics for the selected variables were shown in Table 1. These statistics showed that approximately 35% were younger children and of these, 52.9% were male, also 28.5% were underweight, 44.2% were living in rural areas and 24.9% were the poorest. The trend of prevalence for childhood anemia is presented in Fig 3 below that shows an increase of 6.5% during the years 2011–2016.

**Table 1:** Prevalence of any level of anemia (< 11.0 gm/dl) and moderate to severe anemia among children under 5 years of age (N = 1,942).

Characteristics	Number of Children	Anemia (< 11.0 gm/dl)		Moderate to severe anemia	
	Subject (%)	Prevalence	P values	Subject (%)	P values
**Age of children (in months)**					
0–11	223 (11.5%)	159 (71.6%)	**<0.001**	95 (43.3%)	**<0.001**
12–23	464 (23.9%)	323 (68.9%)		188 (39.0%)	
24–35	415 (21.4%)	205 (50.5%)		101 (24.4%)	
36–47	431 (22.2%)	181 (43.2%)		72 (18.2%)	
48–59	409 (21.0%)	143 (35.9%)		56 (14.2%)	
**Sex of child**					
male	1,027 (52.9%)	541 (53.5%)	**0.564**	289 (28.7%)	**0.060**
female	915 (47.1%)	470 (51.7%)		223 (24.2%)	
**Birth order of child**					
First	735 (37.8%)	367 (50.2%)	**0.279**	177 (24.1%)	**0.204**
Second	567 (29.2%)	297 (51.9%)		157 (26.7%)	
Third & above	640 (33.0%)	347 (55.9%)		178 (29.2%)	
**Child underweight**					
No	1,388 (71.5%)	681 (49.4%)	**<0.001**	338 (24.4%)	**0.001**
Yes	554 (28.5%)	330 (60.7%)		174 (32.0%)	
**Mother's BMI**					
Underweight	372 (19.1%)	222 (60.2%)	**<0.001**	121 (33.5%)	**<0.001**
Normal	1,285 (66.2%)	663 (51.7%)		339 (26.3%)	
Overweight	285 (14.7%)	126 (46.3%)		52 (18.3%)	
**Mother's anemia status**					
Not anemic	1,083 (55.8%)	472 (44.6%)	**<0.001**	216 (20.4%)	**<0.001**
Anemic	859 (44.23%)	539 (62.0%)		296 (33.9%)	
**Mother's educational status**					
No education	664 (34.2%)	379 (57.3%)	**<0.001**	189 (29.8%)	**0.022**
Primary	359 (18.5%)	188 (52.8%)		102 (27.7%)	
Secondary	634 (32.6%)	328 (52.9%)		166 (24.9%)	
Higher	285 (14.7%)	116 (39.1%)		55 (20.4%)	
**Place of residence**					
Rural	1,083 (55.8%)	540 (49.4%)	**0.029**	262 (22.7%)	**0.015**
Urban	859 (44.2%)	471 (56.2%)		250 (30.8%)	
**Socio-economic status**					
Poorest	483 (24.9%)	239 (49.1%)	**<0.001**	120 (22.8%)	**0.006**
Poorer	427 (22.0%)	205 (48.6%)		101 (24.8%)	
Middle	422 (21.7%)	260 (61.9%)		134 (32.7%)	
Richer	386 (19.9%)	219 (57.6%)		112 (29.3%)	
Richest	224 (11.5%)	88 (40.6%)		45 (20.4%)	
**Development Region**					
Eastern	362 (18.6%)	201 (54.9%)	**0.349**	98 (26.2%)	**0.110**
Central	502 (26.0%)	271 (53.1%)		149 (29.3%)	
Western	395 (20.3%)	196 (50.4%)		86 (22.3%)	
Mid-western	420 (21.6%)	209 (51.6%)		107 (25.8%)	
Far western	263 (13.5%)	134 (50.8%)		72 (27.3%)	
**Ecological region**					
Mountain	159 (8.2%)	91 (57.2%)	**<0.001**	50 (31.1%)	**<0.001**
Hill	810 (41.7%)	333 (39.9%)		156 (17.8%)	
Terrain	973 (50.1%)	587 (60.6%)		306 (32.0%)	
**Overall**	1,942 (100.0%)	1011 (52.6%)		512 (26.6%)	

Significant at 5% level

### Prevalence of any anemia and moderate to severe anemia

**Fig 4:**
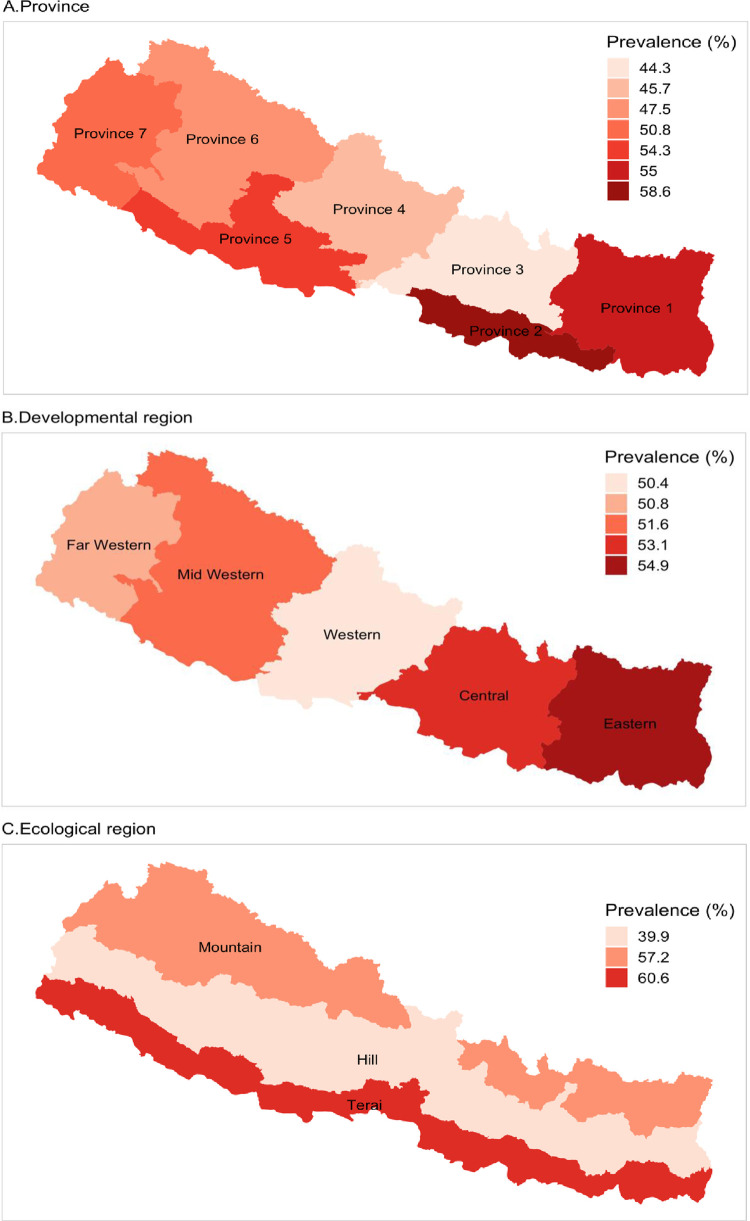
Regional variations in prevalence of any level of anemia among Nepalese children.

**Fig 5:**
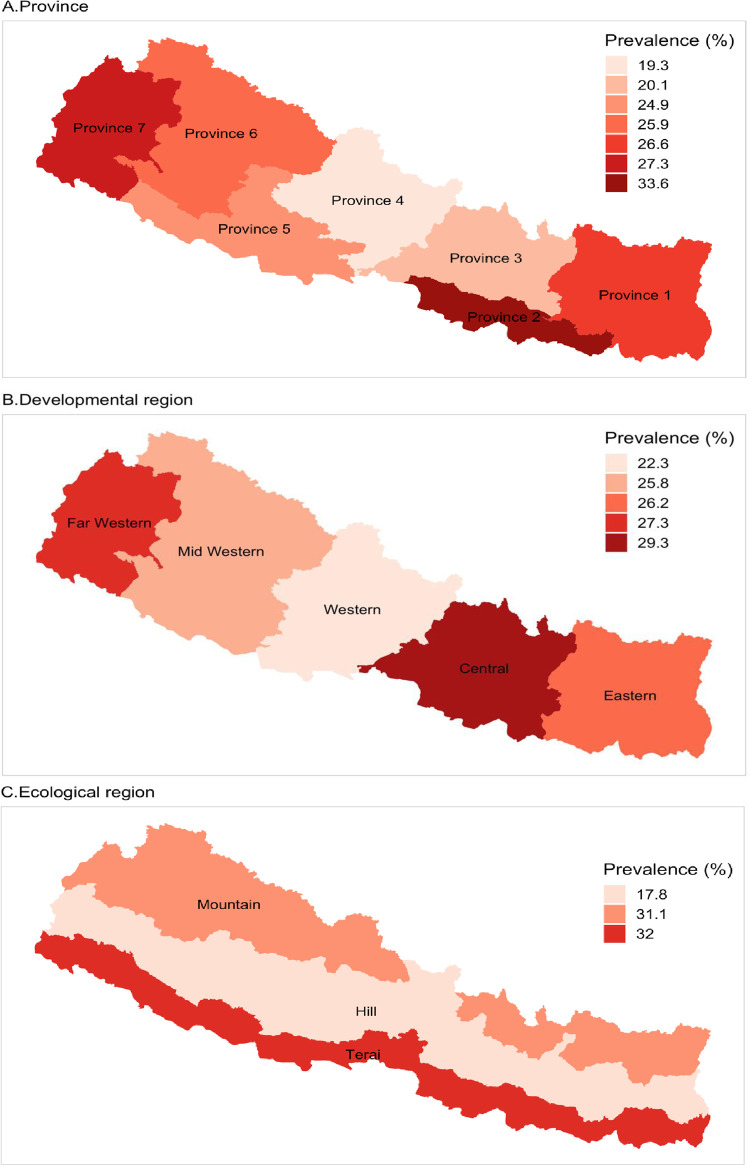
Regional variations in prevalence of moderate to severe anemia among Nepalese children.

Table 1 showed that the prevalence of both any level of anemia (<11.0 gm/dl) and moderate to severe anemia were significantly higher among children aged less than 11 months (71.6% were anemic and 43.3% were moderate to severe anemic) and children of uneducated mothers (57.3% were anemic and 29.8% were moderate to severe anemic). The prevalence of anemia in both cases (e.g., any level of anemia and moderate to severe anemia) was also higher among underweight children (60.7% were anemic and 32% were moderate to severe anemic), children of underweight mothers (60.2% were anemic and 33.5% were moderate to severe anemic) and children of anemic mothers (62% were anemic and 33.9% were moderate to severe anemic). The prevalence of anemia, however, was higher among children from middle class families (61.9% were anemic and 32.7% were moderate to severe anemic) and also higher among children in terrain ecological regions (60.6% were anemic and 32% were moderate to severe anemic). Regional variations in prevalence are presented in Figs 4 and 5 below.

### Factors associated with anemia estimated by multilevel analysis

The results from adjusted multilevel logistic regression analysis presented in Table 2 showed that the model for any level of anemia (<11.0 gm/dl) was statistically significant (likelihood-ratio *x*^2^ = 16.13, p = 0.0003). The effects of variances at community and household levels were also significant for the model.

**Table 2:** Determinants of any level of anemia (< 11.0 gm/dl) among children under 5 years old.

Characteristics	Unadjusted Odds Ratio (95% CI)	p-value	Adjusted Odds Ratio (95% CI)	p-value
**Age of children (in months)**				
< 11	4.62 (3.24–6.59)	<0.001	7.31 (4.37–12.23)	<0.001
12–23	4.26 (3.21–5.66)	<0.001	6.31 (4.14–9.62)	<0.001
24–35	1.82 (1.37–2.40)	<0.001	2.20 (1.54–3.15)	<0.001
36–47	1.35 (1.02–1.78)	0.037	1.47 (1.05–2.05)	0.026
48–59	1.00		1.00	
**Sex of child**				
Male	1.00		1.00	
Female	0.95 (0.79–1.13)	0.564	0.93 (0.74–1.17)	0.530
**Birth order of child**				
First	1.00		1.00	
Second	1.10 (0.89–1.37)	0.381	1.12 (0.86–1.46)	0.415
Third & above	1.18 (0.96–1.47)	0.113	1.01 (0.74–1.38)	0.959
**Child underweight**				
No	1.00		1.00	
Yes	1.53 (1.25–1.87)	<0.001	1.46 (1.11–1.92)	0.007
**Mother's BMI**				
Underweight	1.00		1.00	
Normal	1.39 (1.01–1.76)	0.006	1.07 (0.81–1.43)	0.633
Overweight	0.74 (0.57–0.96)	0.024	1.03 (0.74–1.43)	0.876
**Mother's anemia status**				
Not anemic	1.00		1.00	
Anemic	2.18 (1.82–2.62)	<0.001	1.99 (1.53–2.59)	<0.001
**Mother's educational status**				
No education	1.94 (1.46–2.57)	<0.001	1.80 (1.14–2.86)	0.012
Primary	1.60 (1.17–2.19)	0.003	1.44 (0.89–2.34)	0.141
Secondary	1.56 (1.18–2.07)	0.002	1.55 (1.03–2.34)	0.035
Higher	1.00		1.00	
**Place of residence**				
Rural	1.00		1.00	
Urban	0.82 (0.03–0.68)	0.030	0.88 (0.68–1.16)	0.372
**Socio-economic status**				
Poorest	1.51 (1.10–2.09)	0.012	1.68 (0.99–2.84)	0.055
Poorer	1.43 (1.03–1.98)	0.034	1.25 (0.78–2.00)	0.360
Middle	2.48 (1.78–3.46)	<0.001	1.89 (1.17–3.07)	0.010
Richer	2.03 (1.45–2.83)	<0.001	1.63 (1.03–2.58)	0.039
Richest	1.00		1.00	
**Development Region**				
Eastern	1.06 (0.81–1.40)	0.654	0.95(0.63–1.41)	0.788
Central	1.00		1.00	
Western	0.84 (0.64–1.09)	0.194	1.10 (0.76–1.59)	0.624
Mid-western	0.84 (0.65–1.09)	0.201	1.07 (0.71–1.62)	0.742
Far western	0.89 (0.66–1.19)	0.425	0.88 (0.56–1.38)	0.574
**Ecological region**				
Mountain	1.92 (1.36–2.70)	<0.001	1.93 (1.14–3.3)	0.015
Hill	1.00		1.00	
Terrain	2.18 (1.80–2.63)	<0.001	2.23 (1.57–3.17)	<0.001
**Random effect variance**				
Level 2 (Household)			0.63 (0.41*)	
Level 3 (Community)			0.30 (0.11*)	

Adjusted odds ratios (OR) with 95% confidence interval (CI) were reported from a multilevel logistic regression model accounting for intercept at household and community.

“*” denotes the standard error (SE) of random intercept and it measures the variability of the average effect in each level (community and household) experiencing malnutrition. The P-value for each random effect variance is 0.0003.

The results showed that children aged less than 11 months were more likely to be anemic (Adjusted Odds Ratio (AOR): 7.31, 95% Confidence Interval (CI) = 4.37–12.23; p = <0.001) compared to the 48–59 months age group while explaining the random effect. Underweight children (AOR: 1.46, 95% CI = 1.11–1.92; p = <0.007), children of anemic mothers (AOR: 1.99, 95% CI = 1.53–2.59; p = <0.001) and children of uneducated mothers (AOR: 1.80, 95% CI = 1.14–2.86; p = 0.012) had higher odds of being anemic as did children from middle class families (AOR: 1.89, 95% CI = 1.17–3.07; p = <0.010). In addition, children from ecologically terrain regions were more likely to be anemic (AOR: 2.23, 95% CI = 1.57–3.17; p = <0.001) than children from hill regions.

### Factors associated with moderate to severe anemia

The adjusted multilevel logistic regression analysis results (presented in Table 3) showed the model for moderate to severe anemia that was statistically significant (likelihood-ratio *x*^2^ = 13.21, p = 0.0014). The variances at community and household levels were shown to be significant for the model. Underweight children (AOR: 1.58, 95% CI = 1.05–2.37; p = 0.028), children of anemic mothers (AOR: 2.96, 95% CI = 1.89–4.63; p = <0.001), children whose mothers had completed secondary education (AOR: 2.15, 95% CI = 1.17–3.96; p = 0.014) and those children living in ecologically terrain regions (AOR: 2.95, 95% CI = 1.64–5.31; p = <0.001) had higher odds of developing moderate to severe anemia with regard to random effects. Children aged under 11 months had a higher likelihood of moderate to severe anemia (AOR: 20.81, 95% CI = 7.62–56.80; p = <0.001) compared to children in the oldest age group.

**Table 3:** Determinants of moderate-to-severe anemia among children under 5 years old.

Characteristics	Unadjusted Odds Ratio (95% CI)	p-value	Adjusted Odds Ratio (95% CI)	p-value
**Age of children (in months)**				
< 11	7.05 (4.59–10.82)	<0.001	20.81 (7.62–56.80)	<0.001
12–23	6.33 (4.41–9.09)	<0.001	17.35 (6.96–43.26)	<0.001
24–35	2.28 (1.57–3.32)	<0.001	3.40 (1.85–6.26)	<0.001
36–47	1.37 (0.93–2.02)	0.115	1.56 (0.95–2.56)	0.081
48–59	1.00		1.00	
**Sex of child**				
Male	1.00		1.00	
Female	0.84 (0.68–1.05)	0.122	0.82 (0.59–1.14)	0.232
**Birth order of child**				
First	1.00		1.00	
Second	1.21 (0.93–1.58)	0.162	1.48 (0.95–2.30)	0.083
Third & above	1.26 (0.98–1.64)	0.077	1.23 (0.77–1.98)	0.388
**Child underweight**				
No	1.00		1.00	
Yes	1.62 (0.42–0.54)	<0.001	1.58 (1.05–2.37)	0.028
**Mother's BMI**				
Underweight	1.00		1.00	
Normal	1.48 (1.13–0.84)	0.005	1.19 (0.77–1.84)	0.430
Overweight	0.60 (0.43–0.84)	0.003	0.82 (0.48–1.42)	0.484
**Mother's anemia status**				
Not anemic	1.00		1.00	
Anemic	2.62 (2.10–3.23)	<0.001	2.96 (1.89–4.63)	<0.001
**Mother's educational status**				
No education	2.04 (1.43–2.91)	<0.001	2.11 (1.06–4.23)	0.035
Primary	1.83 (1.24–2.71)	0.002	1.96 (1.01–3.82)	0.047
Secondary	1.67 (1.17–2.38)	0.005	2.15 (1.17–3.96)	0.014
Higher	1.00		1.00	
**Place of residence**				
Rural	1.00		1.00	
Urban	0.75 (0.60–0.93)	0.009	0.79 (0.53–1.17)	0.245
**Socio-economic status**				
Poorest	1.49 (0.99–2.22)	0.053	1.72 (0.75–3.95)	0.204
Poorer	1.37 (0.91–2.07)	0.129	1.17 (0.57–2.40)	0.674
Middle	2.50 (1.66–3.76)	0.001	1.75 (0.88–3.52)	0.113
Richer	2.02 (1.34–3.06)	0.001	1.71 (0.85–3.44)	0.135
Richest	1.00		1.00	
**Development Region**				
Eastern	0.94 (0.68–1.31)	0.726	0.76 (0.41–1.38)	0.361
Central	1.00		1.00	
Western	0.67 (0.48–0.93)	0.016	0.85 (0.48–1.52)	0.580
Mid-western	0.79 (0.58–1.07)	0.129	1.01 (0.56–1.82)	0.983
Far western	0.87 (0.61–1.23)	0.424	0.74 (0.38–1.44)	0.377
**Ecological region**				
Mountain	2.25 (1.50–3.38)	<0.001	2.70 (1.28–5.7)	0.009
Hill	1.00		1.00	
Terrain	2.42 (1.92–3.07)	<0.001	2.95 (1.64–5.31)	<0.001
**Random effect variance**				
Level 2 (Household)			1.98 (1.22*)	
Level 3 (Community)			0.46 (0.27*)	

Adjusted odds ratios (OR) with 95% confidence interval (CI) were reported from a multilevel logistic regression model accounting for intercept at household and community.

“*” denotes the standard error (SE) of random intercept and it measures the variability of average effect in each level (community and household) experiencing malnutrition. The P-value for each random effect variance is 0.0014.

## Discussion

In 2016, the prevalence of childhood anemia in Nepal was reported to be at 52.6%, an indication of a considerable increase after a decline in numbers had been reported between 2006–2011 [20]. Recent DHS records showed that childhood anemia was high in some South and Southeast Asian countries, such as India (58.5% in 2016), Myanmar (57.8% in 2016), Cambodia (55.5% in 2014), Bangladesh (51.3% in 2011), Maldives (49.7% in 2017) and Timor-Leste (40.3% in 2016) plus Nepal [20]. This study suggests that Nepal has not yet achieved sustainable improvement in maternal and child nutrition. Possible reasons for this could be that children in rural areas suffering from undernutrition and ill health may be due not only to poor diet but also because of direct exposure to household air pollution, polluted drinking water, poor hygiene and sanitation. The level of knowledge and understanding of multisector approaches is relatively low in Nepal and are allied to a lack of coordination among key sectors, such as health, agriculture, education, urban development and local development that impacts on their ability to address the issue of nutrition. There is also a lack of communication and coordination between key government sectors and development agencies along with limited budgets allocated from each sector and by donors. Poor coordination between key institutions such as in government, academia, research, training and national/international nongovernmental organizations add to the problems [21]. The inability to scale up action on nutritional activities in all districts is potentially compounded by a number of problems including a lack of research-based evidence, poor planning, diverse services and working cultures, short-term funding, limited technical and coordination expertise, overlapping of services in different sectors leading to duplication and differing health messages, low priority assigned to nutritional needs at all levels and lack of clarity as to how each sector should engage with the issue of nutrition [22]. Significant progress has been observed in Sri Lanka where the current prevalence of anemia among children stands at 26% that may be due to the successful implementation of various strategic plans and programmes that focused on reducing it [1, 23, 24].

The prevalence of moderate to severe childhood anemia in Nepal is reported to be at 26.6%. In 2011 in Bangladesh, it stood at 22.1%, in 2016 in India it was 30.8% and in 2017 in Maldives it was 20.3% [20]. The higher prevalence of childhood anemia indicates that there is a serious problem and this study can help with the process of reviewing child health policy in order to combat it. Food insecurity due to the effects of climate change on agricultural productivity, the continuing global financial crisis and an undermined socioeconomic future may also be having an impact on the present state of childhood anemia in South Asian countries and need to be addressed [25].

This study showed that the prevalence of anemia was higher among children aged under 11 months and in underweight children. This high prevalence in the younger age group was also found in neighboring countries, such as China and Bangladesh [6, 26]. Breastfeeding after six months of age is no longer sufficient, on its own, for a child’s physical and mental development, meaning that foods such as solid and semi-solid products have an important role to play to complement the breast milk. Appropriate complementary feeding practices may help to reduce the chances of children in the younger age groups from developing anemia. Childhood anemia was found to be higher among female children in Bangladesh, Sudan and Brazil [26–28] and also among the third and above birth order children in Indian and Egyptian families [29, 30]. This could possibly be explained by a scarcity of food for sharing among children in larger families. Underweight children in Bangladesh, Pakistan, Ethiopia and India were found to have a higher prevalence of anemia [17, 18, 29, 31]. The reasons for being underweight may be due to poor distribution of inadequate food within the family, food insecurity, poverty and micronutrient deficiencies that often tend to coexist with other macronutrient deficiencies [29]. Overall poverty in Nepal stood at 25% and food insecurity at 23%, with both comparatively higher in mountainous regions followed by hills and terrain regions [32]. In this study, the prevalence of anemia was found to be significantly higher in terrain regions that is potentially due to deprivation and lack of basic education and health related facilities. The ethnic populations in these regions are socially, culturally and economically excluded from mainstream development and experience particular challenges in accessing health, education and other resources [33]. Western terrain regions are less developed with regard to economic infrastructure, job market and transportation and lack of quality farming soil, rainfall and duration of the crop growing season all leading to less productive farming. Lower consumption of iron-rich foods that is associated with childhood anemia has been reported in the terrain regions but not in the hills and mountain regions [34].

The association between maternal circumstances and childhood anemia in Nepal has not been sufficiently researched. This study showed that underweight, anemic and uneducated mothers had a higher prevalence of anemic children. However, in Ethiopia, children of overweight mothers have also been reported as having a higher prevalence of anemia [31]. In Bangladesh, Pakistan and China, a higher prevalence of childhood anemia was noted among anemic mothers [17, 18, 35] and in India, it was found among uneducated mothers [29]. In many societies, women's inferior social status within the household with poor access to assets, employment, health care, and education adversely affects their health and that of their children. In Nepal, a low level of autonomy among women leads to poor health service utilization and has a direct impact on maternal and child morbidity and mortality [36].

This study in Nepal is of significance as it uses nationally representative data to comprehensively investigate the relationship between anemia and a range of indicators at individual, household and community levels. The multilevel regression analysis, that was used to identify random effects, showed that children that were underweight, in a young age group, children of mothers who were anemic themselves and uneducated had a higher risk of being anemic. Other studies conducted in India, Nepal and Kuwait also found these factors to be significant determinants of childhood anemia as did the results of studies conducted in Ghana and Ethiopia and in Brazil and Haiti [29, 31, 37–41]. However, all these studies identified certain factors as fixed effects. The study findings also showed that children from middle class families had a higher risk of anemia, but studies conducted in Nepal, Pakistan and Bangladesh showed that the poor socio-economic status of children was a significant risk factor for anemia [5]. Agriculture, industry, commerce, and tourism are major contributors to Nepal’s economy. Prior to the earthquakes, these sectors also provided the majority of employment opportunities but the aftermath left these productive sectors virtually destroyed. This has had a negative impact on the livelihoods of approximately 2.29 million households and 5.6 million workers in 31 districts [42]. In this study, a significant regional effect was observed due to this situation that is supported by another study conducted in Nepal [5]. It is still the case, however, that no previous studies used household and community level variations to help explain the determinants of childhood anemia.

There are differences in findings of the significant determinants of childhood anemia between a number of studies. For example, a study in Nigeria showed that child’s age is a significant determinant of moderate to severe anemia but socio-economic status was not found to have any association with it [43]. However, a study in Kenya found that socio-economic status did have a significant effect on childhood anemia [44] and in Tanzania, a child’s age and mother’s education were identified as significant determinants [45] that concurs with the findings of this study. In China, a mother’s anemic status and region were found to be significantly related to severe childhood anemia [35] that is also consistent with the findings of this study.

Several barriers that are present in Nepal impede its ability to develop effective anemia prevention and control strategies. These barriers include poverty, illiteracy, low coverage of maternal and child health care facilities, poor quality of health services, chronic food insecurity, ethnicity and geography. In order to improve the nutritional status of vulnerable citizens, the government in Nepal should put forward an integrated nutrition-sensitive social protection system [46]. The adverse consequences of anemia can be tackled by identifying and addressing the local determinants and by development of effective strategies to deal with them [47]. For example, one strategy could be to introduce community based short term and long term sustainable maternal education and awareness programmes, with a focus on feeding practices for infant and young children [23, 48]; another strategy could be to design specific cultural interventions to target the most marginalized subpopulations such as low caste families [13].

This study fills gaps in knowledge relating to social determinants of childhood anemia in Nepal, and its documented evidence can play a major role in spearheading positive change to deal with the challenges. The main strength of this study is the identification of the pervasive relationship between multilevel factors with variation at individual, household and community levels and childhood anemia derived from a large nationally representative dataset. Limitations of the study include the cross-sectional nature of the data that does not enable an assessment to be made of the cause and effect relationship between selected factors and outcomes. In addition, information bias due to self-reporting age, education and household assets of respondents in the national dataset could also limit the findings of this study. The covariates used in this study did not include food frequency or dietary habits, circumstances at birth, immunization status, morbidity and ethnicity. In spite of these limitations, this study provides important evidence-based information that will help public health policy makers in Nepal.

## Conclusion

As this study shows, the prevalence of anemia among children in Nepal is a severe public health issue. Many factors for this situation have been identified (for example, age, birth order of child, underweight child, mother’s BMI, mother’s anemia status, mother’s educational status, socio-economic status and regions) as having significant random effects on childhood anemia at community and household levels. The estimated magnitude of the effects of the selected predictors from this multilevel analysis is higher than the values identified by those studies that concentrated on fixed effects. The significant variation at community level indicates an inequality of access to healthcare at that level.

Present strategies aimed at reducing childhood anemia in Nepal need to be addressed and developed using multi-sectoral and comprehensive approaches. The strategies should include increasing the countrywide coverage and compliance of iron/folate supplementation for women; reducing the burden of parasitic infections (helminth and malaria) among women and children, especially in vulnerable areas and promoting dietary modification and improved maternal and child care practices and services in underprivileged areas, among others. Further in-depth research using a wide range of variables, including gender and urban-rural differences, will help inspire further ingenuity in developing effective population-based strategies to reduce the prevalence of childhood anemia in children under five years of age in Nepal.
